# Induction of stable human FOXP3^+^ Tregs by a parasite‐derived TGF‐β mimic

**DOI:** 10.1111/imcb.12475

**Published:** 2021-06-03

**Authors:** Laura Cook, Kyle T Reid, Elmeri Häkkinen, Brett de Bie, Shigeru Tanaka, Danielle J Smyth, Madeleine PJ White, May Q Wong, Qing Huang, Jana K Gillies, Steven F. Ziegler, Rick M Maizels, Megan K Levings

**Affiliations:** ^1^ Department of Medicine University of British Columbia Vancouver BC Canada; ^2^ BC Children’s Hospital Research Institute University of British Columbia Vancouver BC Canada; ^3^ Department of Translational Research Benaroya Research Institute, Virginia Mason Seattle WA USA; ^4^ Wellcome Centre for Integrative Parasitology Institute of Infection, Immunity and Inflammation University of Glasgow Glasgow UK; ^5^ Department of Surgery University of British Columbia Vancouver BC Canada; ^6^ School of Biomedical Engineering University of British Columbia Vancouver BC Canada; ^7^ Present address: The Peter Doherty Institute for Infection and Immunity The University of Melbourne Parkville VIC Australia; ^8^ Present address: Department of Allergy and Clinical Immunology Graduate School of Medicine Chiba University Chiba Japan; ^9^ Present address: Division of Cell Signalling and Immunology School of Life Science University of Dundee Dundee UK

**Keywords:** host parasite interactions, inflammatory disease, regulatory T cells, transforming growth factor beta

## Abstract

Immune homeostasis in the intestine is tightly controlled by FOXP3^+^ regulatory T cells (Tregs), defects of which are linked to the development of chronic conditions, such as inflammatory bowel disease (IBD). As a mechanism of immune evasion, several species of intestinal parasites boost Treg activity. The parasite *Heligmosomoides polygyrus* is known to secrete a molecule (*Hp‐*TGM) that mimics the ability of TGF‐β to induce FOXP3 expression in CD4^+^ T cells. The study aimed to investigate whether *Hp‐*TGM could induce human FOXP3^+^ Tregs as a potential therapeutic approach for inflammatory diseases. CD4^+^ T cells from healthy volunteers were expanded in the presence of *Hp‐*TGM or TGF‐β. Treg induction was measured by flow cytometric detection of FOXP3 and other Treg markers, such as CD25 and CTLA‐4. Epigenetic changes were detected using ChIP‐Seq and pyrosequencing of *FOXP3*. Treg phenotype stability was assessed following inflammatory cytokine challenge and Treg function was evaluated by cellular co‐culture suppression assays and cytometric bead arrays for secreted cytokines. *Hp‐*TGM efficiently induced FOXP3 expression (> 60%), in addition to CD25 and CTLA‐4, and caused epigenetic modification of the *FOXP3* locus to a greater extent than TGF‐β. *Hp‐*TGM‐induced Tregs had superior suppressive function compared with TGF‐β‐induced Tregs, and retained their phenotype following exposure to inflammatory cytokines. Furthermore, *Hp*‐TGM induced a Treg‐like phenotype in *in vivo* differentiated Th1 and Th17 cells, indicating its potential to re‐program memory cells to enhance immune tolerance. These data indicate *Hp‐*TGM has potential to be used to generate stable human FOXP3^+^ Tregs to treat IBD and other inflammatory diseases.

## Graphical abstract

In this study, we showed a parasite‐derived TGF‐β mimic protein can induce stable FOXP3^+^ Tregs from both naive and pre‐committed human memory CD4^+^ T cells. This protein has potential for use as a novel immunoregulatory therapeutic for the treatment of inflammatory disease.

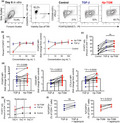

## INTRODUCTION

*Heligmosomoides polygyrus* is a parasitic nematode of mice that establishes long‐term infections in the intestinal tract and releases a diverse array of excretory/secretory products (HES), which promotes parasite survival. Like most parasites, *H*. *polygyrus* has evolved several immune evasion strategies^1^ including those that inhibit inflammatory pathways and promote immunosuppressive cell populations.^2^, ^3^ For example, HES contains molecules that block the cytokine IL‐33 and its receptor ST2,^4^, ^5^ impair dendritic cell function^6^ and suppress macrophages.^7^ Recently, another HES protein was identified as a TGF‐β mimic, termed *Hp*‐TGM (*H. polygyrus* TGF‐β mimic), which, despite the lack of sequence homology, triggered mammalian TGF‐β signaling pathways through binding TGF‐β receptors.^8^ Importantly, *Hp*‐TGM induced FOXP3 expression in both mouse and human CD4^+^ T cells, with mouse induced FOXP3^+^ regulatory T cells (Tregs) shown to have suppressive function both *in vitro* ^8^ and *in vivo*.^9^

FOXP3^+^ Tregs have a key role in maintaining self‐tolerance and immune homeostasis and *in vivo* are generated in both the thymus and the periphery.^10^ Peripheral induction of Tregs in the gut is important to ensure appropriate immune tolerance towards self, commensal and dietary antigens, a setting in which TGF‐β plays a critical role.^11^, ^12^ TGF‐β signaling is tightly regulated, being first produced in a latent form that includes a latency‐associated peptide (LAP), which prevents receptor binding.^13^ One mechanism of generating active TGF‐β is cleavage via the integrin αvβ8, which is expressed by dendritic cells in the gut; active TGF‐β is then able to initiate Treg induction through binding to the heterodimeric surface receptors, TβRI/TβRII, on CD4^+^ T cells.^14^ Receptor binding initiates phosphorylation of SMAD2/3 proteins, which form a complex with SMAD4 that binds to regions in the promoter of *FOXP3* and drive its expression. FOXP3 is the key master transcription factor of Tregs^14^ as it orchestrates the expression of other Treg markers, resulting in the acquisition of immunosuppressive functions.^15^ In contrast to mammalian TGF‐β, *Hp*‐TGM is secreted in an active form and does not require post‐translational processing to activate TGF‐β receptor‐mediated signaling.^16^

The role of TGF‐β in inducing Tregs has been known for over 15 years^17^, ^18^ and much work has been done to investigate whether it could be used to generate large numbers of Tregs *in vitro* for a cell therapy to induce immune tolerance.^15^, ^19^ However, thus far these approaches have been limited in their ability to generate Tregs with phenotypic stability. Previous studies that generated human Tregs *in vitro* (iTregs) with TGF‐β, and with or without combinations of all‐*trans* retinoic acid and/or rapamycin, showed that FOXP3 expression and suppressive function were unstable. Specifically, upon restimulation in the absence of polarizing conditions iTregs lost these features, likely due to the fact that the iTregs did not undergo the epigenetic re‐modeling needed for stable FOXP3 expression.^20^, ^21^

Here we investigated whether *Hp*‐TGM could induce human FOXP3^+^ Tregs and if the induced cells had superior function or stability compared with TGF‐β‐induced Tregs. This knowledge is important to assess the therapeutic potential of this novel parasite‐derived protein.

## RESULTS

### *Hp*‐TGM induces FOXP3^+^ cells with a Treg phenotype from human naïve CD4^+^ T cells

**Figure 1:**
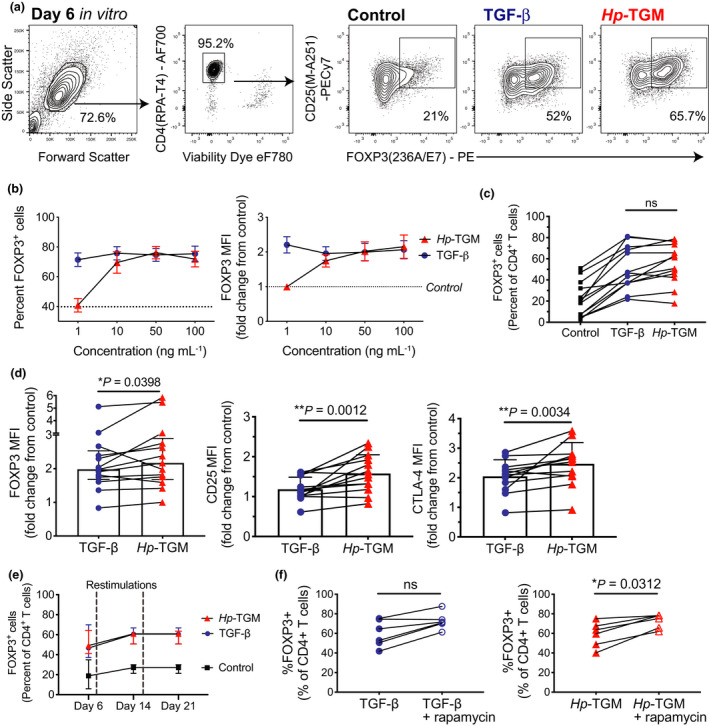
*Hp*‐TGM induces FOXP3^+^ Tregs from naïve human CD4^+^ T cells. Naïve CD4^+^CD8^neg^CD45RA^+^CD25^neg^ T cells were stimulated without or with TGF‐β or *Hp*‐TGM. **(a)** Gating and representative expression of FOXP3 after 6 days culture and **(b)** proportion of FOXP3^+^ cells and FOXP3 MFI across a range of TGF‐β and *Hp*‐TGM concentrations (*n* = 4), median expression in control cultures is indicated by dotted lines. **(c)** Comparison of the percentage of induced FOXP3^+^ cells (*n* = 13) and **(d)** MFI of induced FOXP3, CD25 (*n* = 13) and CTLA‐4 (*n* = 12) in control, TGF‐β and *Hp*‐TGM cultures. **(e)** Percentages of FOXP3^+^ cells were measured at days 6, 14 and 21 (*n* = 7), restimulation time points are indicated by vertical dashed lines. **(f)** Comparison of proportion of FOXP3^+^ cells in cultures with TGF‐β or *Hp*‐TGM without or with rapamycin (*n* = 6). Data in **c–f** used 1 ng mL^−1^ TGF‐β and 100 ng mL^−1^ *Hp*‐TGM. Statistical analyses used Wilcoxon signed rank tests; error bars represent median ± interquartile range, *P ≤ 0.05, ** *P* ≤ 0.01 and ns, not significant.

To test the ability of *Hp*‐TGM vs. TGF‐β to induce Tregs, naïve CD4^+^ T cells were isolated from peripheral blood (Supplementary figure  1a) and stimulated with artificial antigen‐presenting cells expressing CD80 and CD58 and loaded with anti‐CD3 in the absence or presence of the indicated cytokine. The majority of published protocols for TGF‐β‐induction of human iTregs from naïve CD4^+^ T cells use between 1 and 5 ng mL^−1^ of TGF‐β .^21^, ^22^, ^23^ We tested TGF‐β at concentrations from 1 to 100 ng mL^−1^ and confirmed that maximal FOXP3 expression (as determined by both the percentage of positive cells and MFI) had occurred by 1 ng mL^−1^ (dose curve data in Figure 1). This equates to 0.04 nm, as activated TGF‐β exists as a 25 kDa dimer, thus 1 ng mL^−1^ TGF‐β was selected as our comparison condition for the study. We found that after 6 days, in the presence of *Hp*‐TGM or TGF‐β, > 50% of CD4^+^ T cells expressed FOXP3, compared with a mean of 21% expression in their absence (Figure 1a). At least 10 ng mL^−1^ (0.21 nm) of the 46.8 kDa *Hp*‐TGM was required to induce levels of FOXP3 that were equivalent to 1 ng mL^−1^ (0.04 nm) TGF‐β (i.e. ~5.25 times more than TGF‐β) (Figure 1b). Analysis of the dose titration results obtained from all donors, and also in experiments that used anti‐CD3/CD28 bead stimulation (Supplementary figure 1 and data not shown), led us to select 100 ng mL^−1^ *Hp‐*TGM as the concentration that most consistently induced maximal FOXP3 expression and this concentration was used for the remainder of the study.

Although there was no significant difference between TGF‐β and *Hp*‐TGM in terms of the induced proportion of FOXP3‐expressing cells (Figure 1c), there was a small, but significant, increase in FOXP3 MFI in *Hp*‐TGM cultures (Figure 1d). A similar effect was seen for the induction of two Treg markers CD25 (IL‐2Rα) and the co‐inhibitory receptor CTLA‐4, for which MFIs were significantly higher in *Hp*‐TGM cultures compared to those with TGF‐β (Figure 1d). The proportion of induced FOXP3^+^ cells remained consistent upon repeated TCR stimulation and re‐addition of *Hp*‐TGM or TGF‐β (Figure 1e), and in *Hp*‐TGM cultures it could be further enhanced by the addition of rapamycin, the mammalian target of rapamycin (mTOR) inhibitor that suppresses conventional T‐cell proliferation^24^ (Figure 1f).

Similar effects were seen when naïve CD4^+^ T cells were stimulated with anti‐CD3/CD28 beads, although under these conditions the overall proportions of induced FOXP3^+^ cells were lower (~30–40% in TGF‐β/*Hp*‐TGM vs. 10% in control cultures; Supplementary figure  1b, c). Therefore, using two different methods of polyclonal TCR stimulation, *Hp*‐TGM and TGF‐β induced similar proportions of FOXP3^+^ cells, with similar dose‐responses (Supplementary figure  1c), but *Hp*‐TGM induced significantly higher expression of FOXP3, CD25 and CTLA‐4 protein, as measured by MFI. From here on, we refer to the cultures with induced FOXP3^+^ cells as TGF‐β‐ or *Hp*‐TGM‐induced Tregs.

### *Hp*‐TGM signals via TβRI and SMAD2/3 pathways in naïve CD4^+^ T cells

**Figure 2:**
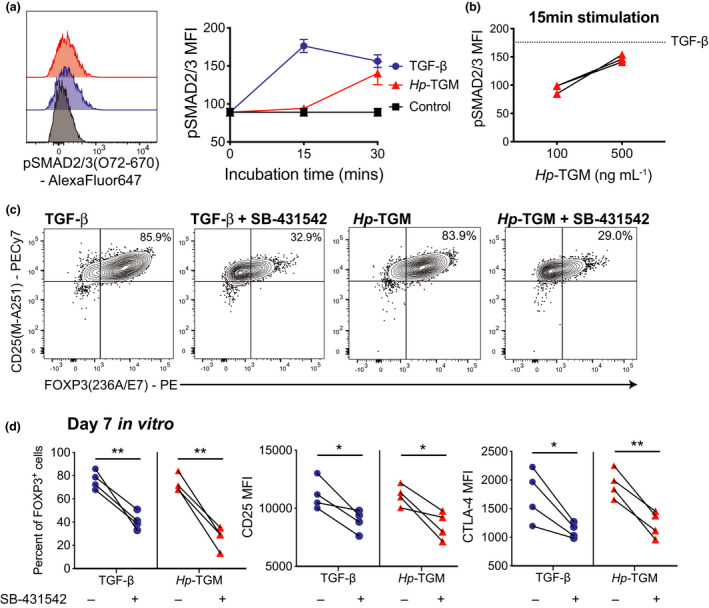
*Hp*‐TGM induces FOXP3 via TGF‐β receptor signaling. **(a)** PBMCs were cultured either alone (control) or with 1 ng mL^−1^ TGF‐β or 100 ng mL^−1^ *Hp*‐TGM for 15 or 30 min and pSMAD2/3 levels assessed by flow cytometry after gating on live CD4^+^ T cells (*n* = 3). **(b)** To assess the dose dependent effect of *Hp*‐TGM, PBMCs were cultured with either 100 or 500 ng mL^−1^ *Hp‐*TGM for 15 min (*n* *= 3)*. **(c, d)** Naïve CD4^+^ T cells were cultured with 1 ng mL^−1^ TGF‐β or 100 ng mL^−1^ *Hp‐*TGM for 5 days with and without addition of the TβRI inhibitor SB‐431542 (5 μm) and the amount of induced FOXP3 was assessed. **(c)** Representative data and **(d)** collated data for *n* = 4 donors from independent experiments. Paired two‐tailed *t*‐tests, error bars represent median ± interquartile range, *P ≤ 0.05, **P ≤ 0.01.

We next asked whether, as seen in mice,^8^ *Hp*‐TGM signaling required the TGF‐β receptor subunit I (TβRI, the serine/threonine kinase ALK5), and stimulated phosphorylation of SMAD2/3 in human cells. PBMCs were cultured in serum‐free media (no exogenous TGF‐β) with either TGF‐β or *Hp‐*TGM and the levels of pSMAD2/3 in CD4^+^ T cells were measured by flow cytometry. TGF‐β rapidly induced pSMAD2/3 after 15 min; stimulation with 100 ng mL^−1^ of *Hp*‐TGM required 30 min to observe a similar effect. However, increasing the concentration of *Hp*‐TGM from 100 to 500 ng mL^−1^ enabled a more rapid activation of pSMAD2/3 (Figure 2a). We confirmed that both *Hp*‐TGM and TGF‐β‐mediated FOXP3‐induction in naïve CD4^+^ T cells required TβRI signaling as inhibition of this receptor with the ALK5 kinase inhibitor SB‐431542 reduced the proportions of FOXP3^+^ cells in both *Hp*‐TGM and TGF‐β conditions by approximately 60% (Figure 2c, d). TβRI inhibition also reduced CD25 and CTLA‐4 expression, with both TGF‐β and TGM cultures having reduced MFIs of approximately 30% (for CD25) and 20% (for CTLA‐4) compared with cultures without inhibitor added (Figure 2c, d).

### *Hp*‐TGM induces epigenetic changes at the FOXP3 locus in naïve CD4^+^ T cells

**Figure 3:**
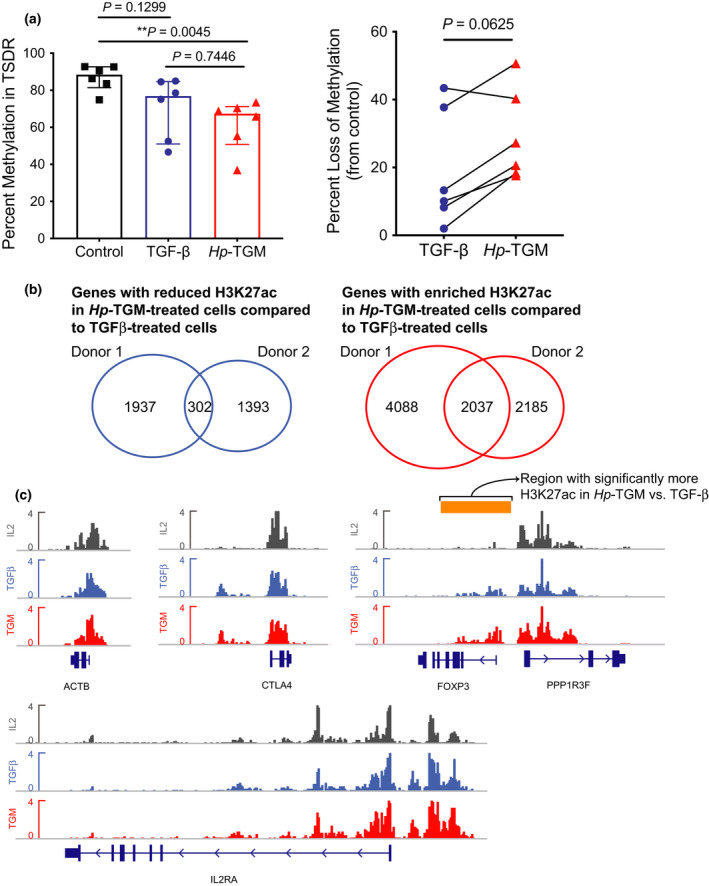
*Hp*‐TGM induces epigenetic modification of *FOXP3*. Naïve CD4^+^ T cells were expanded with modified L‐cells (as per methods) for 28–32 days with either TGF‐β, or *Hp*‐TGM (controls had neither added), then viable CD4^+^ T cells were isolated from cultures by cell sorting. **(a)** The average percentage methylation is shown for eight CpG sites in the TSDR of *FOXP3* for control, TGF‐β and *Hp*‐TGM‐conditioned cells (*n* = 6, all males). Data from TGF‐β and *Hp*‐TGM cultures were converted to a percentage loss of methylation from control. From some cultures, the cells were collected at day 22 in culture, fixed in paraformaldehyde and analyzed by ChIP‐Seq. **(b)** Genes with reduced (left) and enriched (right) H3K27Ac marks in *Hp‐*TGM‐treated cells compared with TGF‐β‐treated cells. Shown are Venn diagrams of genes with reduced/enriched H3K27Ac marks comparing *n* = 2 donors. **(c)** ChIP‐seq of H3K27Ac tracks at *ACTB* (β‐actin, control), *CTLA4*, *IL2RA* (CD25) and *FOXP3* loci. The *FOXP3* region identified by Spatial Clustering for Identification of ChIP‐Enriched Regions (SICER) analysis as having significantly more H3K27ac marks compared with TGF‐β is indicated by the orange bar. Analysis in **a** used Friedman one‐way ANOVA and the Mann‐Whitney *U‐*test and in **b** used the Wilcoxon signed rank test. Error bars represent median ± interquartile range.

As we had observed that *Hp*‐TGM‐induced FOXP3 expression was maintained in culture, we next asked if *Hp*‐TGM caused epigenetic changes in the Treg‐specific demethylated region (TSDR) of the *FOXP3* locus. Reduced methylation in this intronic region is associated with stable FOXP3 expression.^25^ After 28–32 days in culture, *Hp*‐TGM‐ but not TGF‐β‐induced Tregs had significantly less methylation in the *FOXP3* TSDR compared with naïve T cells expanded with only IL‐2 (Figure 3a). We also calculated the percentage loss of methylation for each sample from matched control cultures (100 − (TSDR methylation in *Hp*‐TGM culture/TSDR methylation in control × 100)), which was greater in *Hp*‐TGM cultures than TGF‐β cultures (*P* = 0.0625; Figure 3a).

To further explore possible epigenetic effects of *Hp*‐TGM, we carried out ChIP‐Seq analysis to measure changes in H3K27‐acetylation (ac) on histones, with higher levels being indicative of more active/accessible genes.^26^ Overall, the chromatin modifications detected in TGF‐β‐ vs. *Hp*‐TGM‐induced Tregs were similar, although we did identify genes with different H3K27ac patterns (Figure 3b, c). GO‐enrichment analysis of genes with increased H3K27ac in *Hp*‐TGM‐ compared with TGF‐β‐induced Tregs revealed 58 enriched terms, mostly related to immune functions and modulation of responses by symbionts (Supplementary figure 2). Examples of genes with increased H3K27ac marks in *Hp*‐TGM‐induced compared with TGF‐β‐induced Tregs included the gut homing marker integrin β7 (*ITGB7*), *TGFB*, and its receptor *TGFBR1*, suggesting that *Hp*‐TGM may promote a positive feedback loop of TGF‐β signaling pathways (see GEO series accession number GSE164548). Although both TGF‐β and *Hp*‐TGM induced similar levels of H3K27ac in *CTLA4* and *IL2RA* (CD25), *FOXP3* had a significantly increased number of H3K27Ac tags in *Hp*‐TGM‐ compared with TGF‐β‐induced Tregs as determined by Spatial Clustering for Identification of ChIP‐Enriched Regions (SICER) analysis; the region SICER identified with increased marks is shown by an orange bar in Figure 3c. The latter finding is consistent with the higher MFI of FOXP3 induced by *Hp*‐TGM.

### *Hp*‐TGM‐induced Tregs are functionally suppressive

**Figure 4:**
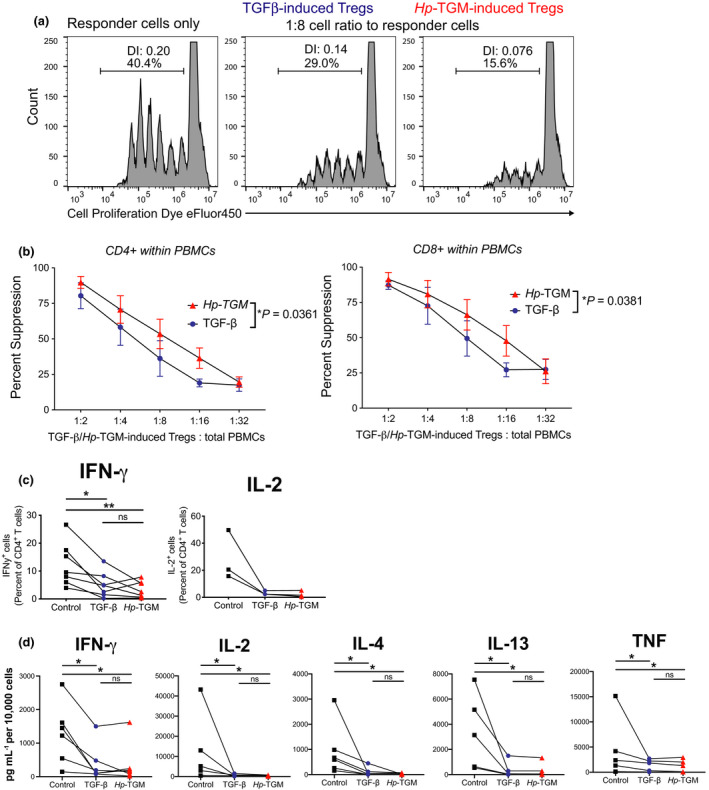
*Hp*‐TGM‐induced FOXP3^+^ cells acquire Treg‐defining functions. **(a)** *In vitro* suppression assays were performed by culturing cell proliferation dye (CPD) eFluor450‐labeled PBMCs (responder cells) with CPDeFluor670‐labelled cells from control, TGF‐β or *Hp*‐TGM cultures 5–7 days after second restimulation at the cell ratios indicated. After 4 days of anti‐CD3/CD28 stimulation, proliferation of responder cells was assessed by calculating division index (DI) and calculating the percentage suppression based on responder only wells. **(b)** The percentage of suppression of responder cells that were gated as live CD4^+^ or CD8^+^ T cells (*n* = 3). Cells from control, TGF‐β or *Hp*‐TGM cultures 5–7 days after second restimulation were cultured with PMA and Ionomycin with or without brefeldin A for 5 h and **(c)** percentages of IFN‐γ^+^ (*n* = 7) and IL‐2^+^ (*n* = 3) cells were measured by flow cytometry (from cultures with brefeldin A added) and **(d)** levels of secreted IFN‐γ, IL‐2, TNF, IL‐4 and IL‐13 were measured in supernatants (from cultures without brefeldin A) by cytometric bead array (*n* = 6; IL‐13 is *n* = 5). A two‐way repeated measures ANOVA was used in (**b** and **a**) Friedman one‐way ANOVA with Dunn’s post‐test were used in **c** and **d**, error bars represent median ± interquartile range, *P ≤ 0.05, **P ≤ 0.01 and ns, not significant.

Next, we investigated whether *Hp*‐TGM‐induced Tregs acquired suppressive activity. We found that, compared with TGF‐β‐induced Tregs, *Hp*‐TGM‐induced Tregs were significantly better at suppressing allogeneic, polyclonally activated CD4^+^ and CD8^+^ T‐cell proliferation over a range of cell ratios (Figure 4a, b). Another defining feature of FOXP3^+^ Tregs is their low expression of effector T‐cell cytokines.^27^ We confirmed that, similar to TGF‐β‐induced Tregs, *Hp*‐TGM‐induced Tregs had significantly reduced secretion of IFN‐γ and IL‐2, compared with control culture cells as detected by intracellular cytokine staining (Figure 4c) and cytokine secretion (Figure 4d). Analysis of the cytokines secreted into culture supernatants also identified both *Hp*‐TGM‐ and TGF‐β‐induced Tregs had significantly reduced secretion of IL‐4, IL‐13 and TNF compared with control cultures (Figure 4d).

### *Hp*‐TGM‐induced FOXP3^+^ Tregs are stable in the presence of inflammatory cytokines

**Figure 5:**
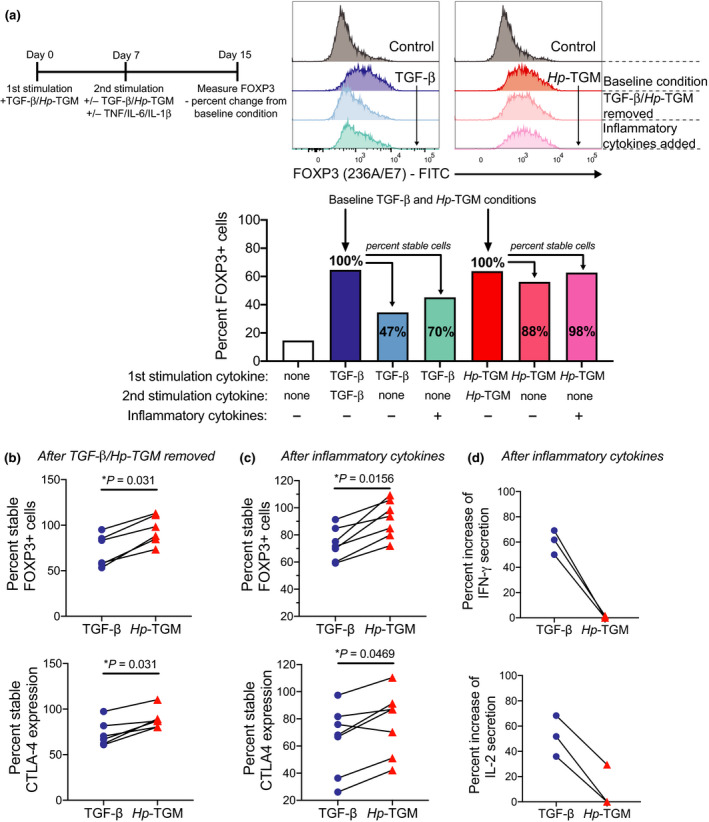
*Hp*‐TGM‐induced FOXP3^+^ Tregs are stable in an inflammatory environment *in vitro*. **(a)** Timeline of how experiments were done to assess stability of induced FOXP3 expression when polarizing cytokines were removed and when inflammatory cytokines (IL‐6, IL‐1β and TNF) were added to the cultures. Representative histograms show changes in FOXP3 expression in TGF‐β or *Hp*‐TGM and control cultures. Bar graph shows representative data indicating how percent stable cells were calculated using baseline cultures. **(b, c)** Collated data showing the percent stable FOXP3^+^ cells when the indicated polarizing cytokines were removed, calculated in relation to baseline conditions which had TGF‐β or *Hp*‐TGM present throughout the entire culture (*n* = 6) and **(c)** when polarizing cytokines were removed and inflammatory cytokines were added (*n* = 7). **(d)** For *n* = 3 of the experiments shown in **c**, we took equivalent cell numbers from all cultures, washed and resuspended in cell culture media that had no additional cytokines added then stimulated for 6 h with PMA/Ionomycin. Changes in the secretion of IFN‐γ and IL‐2 were measured in supernatants using cytometric bead array. Wilcoxon signed rank tests are used in **b** and **e**; Friedman one‐way ANOVA with Dunn’s post‐test was used in **c** and **d**.

A concern regarding the use of *in vitro*‐induced Treg cells for therapy is reversion to an effector T‐cell phenotype *in vivo*.^19^ To investigate the stability of *Hp*‐TGM‐induced Tregs, we re‐cultured induced Tregs in the absence of TGF‐β or *Hp*‐TGM and, in some cultures, in the presence of inflammatory cytokines (Figure 5a). Changes in cell phenotype were compared with populations that were expanded in the continual presence of TGF‐β or *Hp*‐TGM (baseline condition), which were defined as having 100% stability and used to calculate the percentage of stable cells in matched comparison cultures. The proportion of *Hp*‐TGM‐induced Tregs that remained FOXP3^+^ after *Hp*‐TGM removal was significantly greater than for TGF‐β‐induced Tregs when TGF‐β was removed, with a similar result for CTLA‐4 expression (Figure 5b). Moreover, when the induced Tregs were re‐cultured without *Hp*‐TGM/TGF‐β, but with the inflammatory cytokines IL‐6, TNF and IL‐1β, *Hp*‐TGM‐induced Tregs showed significantly greater stability of FOXP3 and CTLA‐4 expression than did TGF‐β‐induced Tregs (Figure 5c). Importantly, following exposure to inflammatory cytokines, TGF‐β‐ but not *Hp*‐TGM‐induced Tregs increased production of IFN‐γ and IL‐2 compared with cultures with continual presence of TGF‐β/*Hp*‐TGM and no inflammatory cytokines (Figure 5d).

### *Hp*‐TGM can induce stable FOXP3^+^ Tregs from pre‐committed memory Th cells

**Figure 6:**
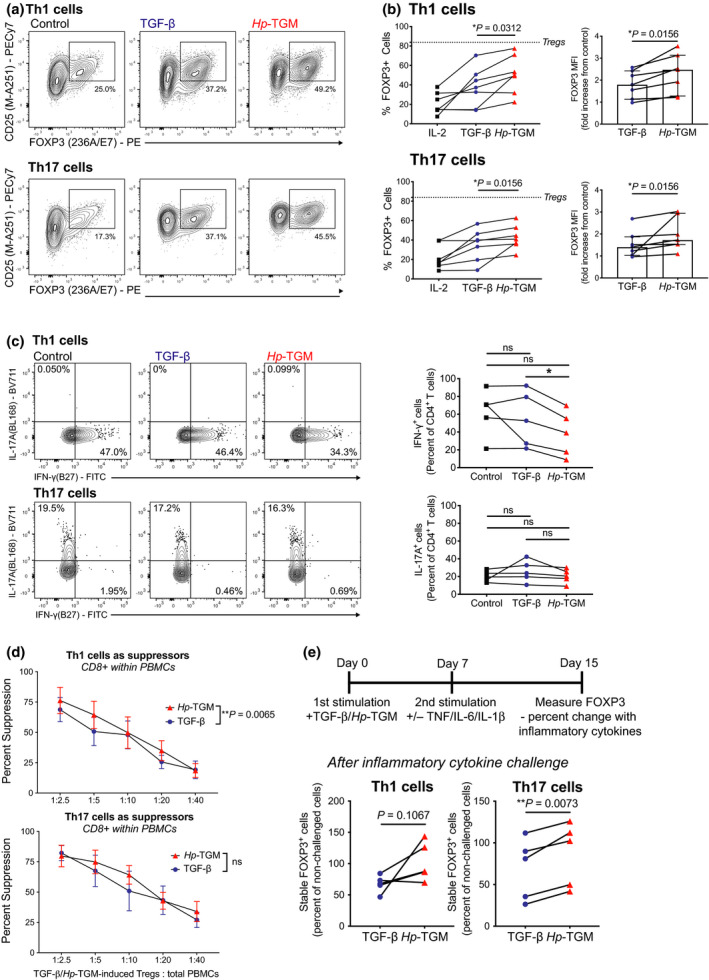
*Hp*‐TGM can induce FOXP3 expression in *ex vivo* memory Th1 and Th17 cells. *Ex vivo* Th cell subsets were isolated by cell sorting as shown in Supplementary figure 3a and expanded with anti‐CD3/CD28 beads (1 bead to 4 cell ratio) alone (control) or with TGF‐β or *Hp*‐TGM. Representative plots and proportion of FOXP3^+^ cells and FOXP3 MFI are shown after 7 days culture of **(a)** Th1 cells and **(b)** Th17 cells (*n* = 7). **(c)** For *n* = 5, changes in IFN‐γ and IL‐17A production were assessed by flow cytometry by restimulating with PMA and ionomycin in the presence of brefeldin A (*n* = 5). **(d)** At day 28 (13 days after second restimulation) suppression assays were performed and the percentage suppression of CD8^+^ responder T cells (within PBMCs) is shown for TGF‐β‐ or *Hp‐*TGM‐conditioned Th1 and Th17 cells (*n* = 4). **(e)** Changes in FOXP3 expression when inflammatory cytokines (IL‐6, IL‐1β and TNF) were added to cultures compared with cultures without cytokine challenge (*n* = 5), * *P* ≤ 0.05 and ns, not significant.

As a potential therapeutic application of *Hp*‐TGM is its direct administration *in vivo*, we investigated whether it may be able to convert pre‐committed memory Th subsets to a regulatory T‐cell phenotype. We sorted *ex vivo* Th1, Th2 and Th17 cells using the gating strategy in Supplementary figure 3a and expanded these cells by stimulating with anti‐CD3/CD28 beads in the absence or presence of TGF‐β or *Hp‐*TGM (Figure 6a). *Hp‐*TGM‐induced significantly more Th1 and Th17 cells to express FOXP3 after 7 days in culture compared with TGF‐β (Figure 6b), although both were similarly unable to induce FOXP3 expression in Th2 cells (Supplementary figure 3b). However, in contrast to what was found with naïve CD4^+^ T‐cell cultures, neither TGF‐β nor *Hp‐*TGM‐conditioned Th1 or Th17 cells had substantial upregulation of CD25 or CTLA‐4 compared to control cultures (Supplementary figure  3c). Interestingly, *Hp‐*TGM, but not TGF‐β, resulted in a small, yet significant, reduction in the percentage of IFN‐γ^+^ Th1 cells and the amount of secreted IFN‐γ. Nevertheless, neither *Hp‐*TGM nor TGF‐β affected IL‐17A expression by Th17 cells (Figure 6c and Supplementary figure 3d). Both TGF‐β and *Hp*‐TGM‐conditioned Th1 and Th17 cells acquired *in vitro* suppressive function, with *Hp*‐TGM‐conditioned Th1 cells being slightly more suppressive than their TGF‐β counterparts over a range of cell ratios (*P* = 0.0065; Figure 6d). Finally, we assessed the stability of the induced Tregs from Th1 and Th17 cell cultures. Intriguingly, we again noted that the *Hp*‐TGM‐induced FOXP3^+^ cells had enhanced stability compared with TGF‐β‐induced FOXP3^+^ cells in the presence of inflammatory cytokines *in vitro*, and this was significant for the Th17 cells (*P* = 0.0073, Figure 6e).

## DISCUSSION

We show here that a novel parasite‐derived TGF‐β mimic protein (*Hp*‐TGM) is able to induce FOXP3^+^ Tregs from both naïve and memory human CD4^+^ T cells *in vitro*. The FOXP3^+^ Tregs induced from naïve CD4^+^ T cells also had high expression of the Treg markers CD25 and CTLA‐4, did not secrete effector cytokines and were able to suppress T‐cell proliferation. Most striking was our finding that, in comparison with TGF‐β, *Hp*‐TGM‐induced Tregs had superior stability *in vitro* in the presence of inflammatory cytokines. These data suggest that *Hp*‐TGM not only phenocopies the ability of TGF‐β to induce Tregs but that these *Hp*‐TGM‐induced Tregs may be a more stable cell product with potential therapeutic benefits.

*Hp*‐TGM was similar to TGF‐β in its ability to induce FOXP3 expression in naïve CD4^+^ T cells; however, comparable levels of FOXP3 induction required at least 5 times higher molar concentrations of *Hp*‐TGM compared with TGF‐β, the opposite of what was previously shown for murine T cells.^7^ The source of *Hp*‐TGM, *H*. *polygyrus*, is a mouse parasite and these data may reflect host–parasite co‐evolution and a specialized adaptation of *Hp*‐TGM to the mouse immune system. As murine and human TGF‐β receptors share 96% amino acid identity, the discordant concentration of *Hp‐*TGM required for its effects on mouse and human T cells is unlikely to be due to differing affinities for the TGF‐β receptors. However, we have shown that a truncated form of *Hp‐*TGM, which retains the receptor binding domains but lacks the C‐terminal portion of the protein, also requires higher concentrations to be effective on mouse cells,^16^ implying that there may be an additional, mouse‐specific, interaction that enhances the efficacy of *Hp*‐TGM.

Although the percentages of FOXP3^+^ cells induced by the two ligands were similar, *Hp*‐TGM‐induced Tregs expressed higher amounts of FOXP3 protein, as indicated by MFI. In a parallel study,^9^ we studied the kinetics of *Hp*‐TGM signaling in mouse T cells and found it had a more sustained effect compared with TGF‐β. This may be due to TGF‐β being subjected to intricate regulation, which *Hp*‐TGM may be able to circumvent, resulting in more prolonged signals and increased amounts of FOXP3. Importantly, we also showed that, compared to TGF‐β, *Hp*‐TGM could induce significantly more expression of CTLA‐4, a co‐inhibitory receptor with an important functional role in Tregs.^28^ The *in vitro* effects of *Hp*‐TGM could also be achieved with anti‐CD3/CD28 bead‐based stimulation, with FOXP3^+^ cell purity further enhanced by rapamycin, suggesting that *Hp‐*TGM could be adapted for induction of a human Treg cell therapy product. Since not all cells were converted to Tregs, more work is needed to understand the biological differences between naïve CD4^+^ T cells that are, or are not, susceptible to the effects of *Hp‐*TGM.

It is remarkable that, despite sharing no sequence homology with mammalian TGF‐β, *Hp*‐TGM is able to signal through the human TGF‐β receptor complex. Of note, one key difference is that *Hp*‐TGM is able to directly bind to TβRI, while TGF‐β must first form a complex with TβRII,^8^ although the functional consequences of this difference are not yet known. We and others have shown that *H. polygyrus* infection induces host TGF‐β production,^29^, ^30^ which may augment the effects of *Hp*‐TGM *in vivo*. However, as *Hp*‐TGM does not require processing for biological activity and is not subject to feedback controls that might limit the effects of TGF‐β it is likely to have a more prolonged and potent effect *in vivo*.

We confirmed that *Hp*‐TGM is able to drive SMAD2/3 phosphorylation in primary human cells, although it required either a longer incubation time or a higher concentration to achieve the same induction of FOXP3 as did TGF‐β. While the mature form of TGF‐β consists of a single domain, *Hp*‐TGM consists of five domains and, in mice, only the first three domains were required for TGF‐β receptor binding and FOXP3 induction.^16^ A recent study of *Hp‐*TGM binding to human receptors identified that domain 3 competes for the same binding sites as TGF‐β on TBRII, while domains 1 and 2 are required for optimal binding to TBRI, with the role of domains 4 and 5 in full‐length *Hp‐*TGM remaining unclear.^31^ It is important for future studies to understand which domains are required for *Hp*‐TGM signaling in human cells, to solve the crystal structure of *Hp*‐TGM and TβRI/II, and determine if *Hp*‐TGM is able to bind other receptors. These data will provide clues as to how TGF‐β signaling pathways could be targeted therapeutically.

Supporting the finding that *Hp*‐TGM induced higher FOXP3 MFI was the fact that *Hp*‐TGM induced a greater loss of methylation in the *FOXP3* TSDR compared with TGF‐β‐induced Tregs, although neither TGF‐β nor *Hp*‐TGM achieved more than a 50% loss of methylation in naïve CD4^+^ T cells. While it is accepted that most thymically derived Tregs have a fully demethylated TSDR, it is still unclear whether this is the case for peripherally derived Tregs and evidence to date suggests that Tregs induced *in vitro* with TGF‐β do not acquire this signature.^20^, ^21^ Interestingly, a recent study in mice reported that TGF‐β and IL‐2 *in vitro*‐induced Tregs acquired a demethylated TSDR in the absence of CD28 signaling.^32^ Future studies should investigate the epigenetic and functional effects on Tregs induced with *Hp‐*TGM without CD28 co‐stimulation. However, as recent studies of iTregs generated by TGF‐β and all‐*trans* retinoic acid and rapamycin^22^; or TGF‐β and small molecule inhibitors^23^; or lentiviral transfer of FOXP3^33^; have found that the iTregs had robust and stable suppressive function without acquiring a methylated TSDR, this feature may not be essential for suppressive function of iTregs. Nevertheless, our data showing increased H3K27ac marks in the *FOXP3* locus in *Hp*‐TGM‐induced Tregs suggest that this molecule may drive epigenetic changes that promote Treg stability^34^ and warrant further investigation.

Importantly, *Hp*‐TGM‐induced FOXP3^+^ Tregs acquired robust *in vitro* suppressive function, which was statistically significantly superior to that of TGF‐β‐induced Tregs. These cells also adopted the typical Treg characteristic of minimal production of effector cytokines, particularly IL‐2, which is a key distinguishing feature from non‐Tregs. Therefore, the *Hp*‐TGM‐induced Tregs fit the criteria proposed by Yamaguchi *et al*. for *bona fide* Tregs, being high, constitutive expression of FOXP3, CD25 and CTLA‐4 and no IL‐2 expression.^28^ To more rigorously assess the stability of the *Hp*‐TGM‐induced Tregs we challenged these cells by removing *Hp*‐TGM and adding the inflammatory cytokines IL‐6, TNF and IL‐1β. In this setting of inflammatory challenge, we saw a marked difference between *Hp*‐TGM​‐ and TGF‐β‐induced Tregs, with the former having significantly greater stability of FOXP3 and CTLA‐4 expression without secretion of IFN‐γ or IL‐2.

We also showed that *Hp*‐TGM is able to similarly induce stable and suppressive FOXP3^+^ cells from memory Th1 and Th17 cells. Interestingly, *Hp*‐TGM‐treated Th1 cells had reduced IFN‐γ secretion, whereas in parallel assays *Hp*‐TGM‐treated Th17 cells did not have altered IL‐17A expression, indicating that *Hp*‐TGM may be best suited for modulating the activity of Th1 cells. There is much interest in therapies that can convert pathogenic effector T cells into Tregs, and these data suggest that *Hp‐*TGM may be a suitable candidate for such an *in vivo* approach.

In conclusion, we extend upon earlier work describing this novel parasite‐derived TGF‐β mimic, *Hp*‐TGM, showing that it is also able to induce populations of suppressive FOXP3^+^ Tregs *in vivo* from both naïve and memory human CD4^+^ T cells via TβRI and pSMAD2/3 signalling. These cells had superior stability compared to TGF‐β‐induced Tregs in an inflammatory milieu. Our data identify *Hp*‐TGM as a potentially useful therapeutic molecule for the treatment of inflammatory disease, such as inflammatory bowel disease.

## METHODS

### Subjects and samples

Study protocols were approved by Clinical Research Ethics Boards of the University of British Columbia (H18‐02553) and Canadian Blood Services (REB 2015.028). Peripheral blood cells from *n* = 28 healthy volunteers (*n* = 11 female, *n* = 17 male) were used.

### Isolation of CD4^+^ T cells

CD4^+^ T cells were isolated from buffy coats (~50 mL) by incubation with 750 μL RosetteSep human CD4^+^ T‐cell enrichment cocktail (STEMCELL Technologies Inc., Vancouver, BC, Canada) for 20 min followed by centrifugation over Ficoll‐Paque (STEMCELL Technologies Inc.). Naïve CD4^+^ T cells were then isolated by cell sorting. The cells were stained for 20 min with fixable viability dye eFluor780 (eBioscience, San Diego, CA, USA), CD4 (RPA‐T4)‐AF700, CD25 (M‐A251)‐PECy7, CD45RO(UCHL1)‐PE, CD127 (HIL‐7R‐M21)‐APC and CD45RA (HI100)‐FITC (BD Biosciences, Franklin Lakes, NJ, USA). Naïve cells were sorted as live CD4^+^CD25^neg^CD45RA^+^CD45RO^neg^ cells, if required Tregs were sorted as controls as live CD4^+^CD25^++^CD127^low^ (Supplementary figure  1a). For some experiments, naïve CD4^+^ T cells were isolated using an EasySep human naïve CD4^+^ T‐cell isolation kit according to manufacturers’ instructions (STEMCELL Technologies Inc.). Memory Th subsets were isolated by staining for 20 min with fixable viability dye eFluor780, CD4 (RPA‐T4)‐FITC, CD127 (HIL‐7R‐M21)‐PE, CD25‐PECy7 (BD Biosciences), CD45RA (2H4LDH11LDB9)‐ECD (Beckman Coulter, Brea, CA, USA), CXCR3 (G025H7) ‐BV421, CCR6 (G034E3)‐APC, CCR4 (L291H4)‐BV605 (BioLegend, San Diego, CA, USA). Th1 cells were isolated as viable CD4^+^CXCR3^+^CCR4^neg^CCR6^neg^, Th2 cells as viable CD4^+^CXCR3^neg^CCR4^+^CCR6^neg^ and Th17 as viable CD4^+^CXCR3^neg^CCR4^+^CCR6^+^ (Supplementary figure 3a).

### Reagents

Recombinant full length *Hp*‐TGM was produced as described previously^8^ and used at 100 ng mL^−1^ unless otherwise stated. Recombinant mammalian TGF‐β1 (R&D Systems Inc. Minneapolis, MN, USA; catalogue number 240‐B) was used at 1 ng mL^−1^ unless otherwise stated. Recombinant human IL‐2 (Proleukin; Prometheus Laboratories, San Diego, CA, USA) was added to Treg‐induction cultures at 100 U mL^−1^, and in cultures of *ex vivo* Tregs at 1000 U mL^−1^. The TβRI (ALK5) inhibitor SB‐431542, which also inhibits ALK4 and ALK7, was used at 5 μM (Tocris Bioscience, Bristol, UK).

### *Invitro* induction of FOXP3^+^ Tregs

Sorted cells were expanded with anti‐CD3/CD28 Dynabeads at specified ratios (Invitrogen, Carlsbad, CA, USA) or irradiated, modified L‐cells at a 1:1 ratio. The modified L cells used were a mouse fibroblast line virally transfected with the human adhesion molecule CD58 (to bind CD2 on target cells and to stabilize the cell–cell interaction), Fc receptor CD32 (to bind the Fc receptor of the CD3 monoclonal antibody in culture to provide T‐cell receptor stimulation), and the costimulatory ligand CD80.^35^ L‐cells were irradiated 50 Gy and 20 000 cells were plated in flat‐bottom 96‐well plates and 20 000 sorted CD4^+^ T cells added. Cytokines were added at specified concentrations and the cells incubated at 37°C (5% CO_2_). T cells were restimulated every 14 days. The culture media for all experiments was X‐VIVO 15 (Lonza, Basel, Switzerland) supplemented with 5% heat‐inactivated human serum (NorthBio Inc, Toronto, ON, Canada), 1% Glutamax and 1% Penicillin‐Streptomycin (Invitrogen). Cell proliferation was measured by staining with 5 μm cell proliferation dye eF450 (Invitrogen). The inflammatory cytokine challenge was the addition of 10 ng mL^−1^ each of recombinant IL‐1β, IL‐6 (STEMCELL Technologies Inc., #78034; #78148) and TNF (eBioscience, #14‐8329‐63). In some experiments, 100 ng mL^−1^ rapamycin was added (Sigma‐Aldrich, St Louis, CA, USA).

### Phenotype analysis by flow cytometry

Cells were stained with 1:1000 dilution of cell viability dye eFluor780 (eBioscience) then fixed and permeabilized with a FOXP3 buffer kit according to the manufacturer’s instructions (BD). The cells were then stained with a mAb cocktail for 15 min before being washed and data acquired on a 4‐laser Fortessa X20 cytometer (BD). Antibodies used in this study were anti‐CD3(UCHT1)‐V500, CD4(RPA‐T4)‐AF700, IFN‐γ(B27)‐FITC, CD127(HIL‐7R‐M21)‐PE or APC, CD25(M‐A251)‐PECy7 or BV711 (BD Biosciences), CD45RA(HI100)‐ECD (Beckman Coulter), CD45RΑ(2H4LDH11LDB9)‐FITC (eBioscience), CD8(OKT8)‐PerCPeF710, FOXP3(236A/E7)‐PE or FITC (eBioscience), CTLA‐4(BNI3)‐BV786, CXCR3(G025H7)‐BV421, CCR4(L291H4)‐BV605, CCR6(G034E3)‐APC, CD45RO(UCHL1)‐PE and IL‐17A(BL168)‐BV711 (BioLegend). For analysis of phosphorylated SMAD2/3, PBMCs were washed twice in serum‐free media, Iscove's Modified Dulbecco's Medium (IMDM, Gibco, Thermo Fisher Scientific Inc, Waltham, MA, USA) supplemented with 1% fetal calf serum (NorthBio Inc). The cells were incubated in this media and either left unstimulated or 5 ng mL^−1^ TGF‐β or 100–500 ng mL^−1^ *Hp‐*TGM were added for 15 or 30 min at 37ºC and all cells were mixed by pipetting (1 × 10^5^ cells in 500 μL). An equal volume of pre‐warmed (to 37ºC) Cytofix (BD) was added to the cells and incubated at 37ºC for 10 min before adding 100 μL of ice‐cold phosphate buffered saline (Thermo Fisher Scientific Inc) and then stained with pSMAD2/3(O72‐670)‐AF647 (BD).

### Suppression assays

Suppression assays were performed as described previously^36^ using heterologous PBMCs as responder cells and measuring the suppression of anti‐CD3/CD28 bead stimulated cell proliferation.

### Cytokine analysis

Cell supernatants were collected following 5 h stimulation with 10 ng mL^−1^ phorbol 12‐myristate 13‐acetate (PMA) and 500 ng mL^−1^ ionomycin in the presence of 10 μg mL^−1^ Brefeldin A (Sigma‐Aldrich). The concentrations of secreted cytokines in the cell supernatants were measured using the LEGENDplex™ 13‐plex Th cytokine bead array kit according to manufacturer’s directions (BioLegend). CBA data were acquired on a 3‐laser Cytoflex cytometer (Beckman Coulter) and analyzed using FCAP array v3 software (BD).

### Treg‐specific demethylation region

Methylation levels within the Treg‐Specific Demethylation Region (TDSR) of *FOXP3* was assessed by pyrosequencing as described previously.^37^

### ChIP‐Seq

Naïve CD4^+^ T cells were expanded with modified L‐cells (as described above) for 22 days with either TGF‐β, or *Hp*‐TGM (controls had neither added), then viable CD4^+^ T cells were isolated from cultures by cell sorting for ChIP‐seq analysis. The cells were fixed with 1% paraformaldehyde (ThermoFisher Scientific Inc), incubated for 10 min, then a 1/10 volume of 1.5 m glycine (Sigma‐Aldrich) was added for 5 min while rotating. The cells were centrifuged then washed twice with 1 mL ice cold PBS and the cell pellets flash frozen in LN_2_ and stored at −80°C. ChIP‐Seq was performed as previously,^38^ anti‐H3K27Ac polyclonal antibody ab4729 was from Abcam (Cambridge, UK). The obtained sequences were mapped to hg19 using Bowtie 2 (v2.1.1).^39^ Uniquely mapped reads were selected and then duplicated reads were discarded. Peak calls were performed using SICER (v1.1.1)^40^ with the threshold of FDR less than 0.000000001. For further analyses and visualization, ChIPPeakAnno (v3.5)^41^ and Integrative Genome Viewer (v2.3.91)^42^ were used. These data have been deposited in NCBI’s Gene Expression Omnibus^43^ and are accessible through GEO Series accession number GSE164548. (https://www.ncbi.nlm.nih.gov/geo/query/acc.cgi).

### Statistics

Statistical analyses between two groups used the Mann‐Whitney *U*‐test or, for paired samples, the Wilcoxon signed rank test. Analysis of ≥ 3 groups used Kruskal‐Wallis one‐way ANOVA or, for paired samples, a Friedman one‐way ANOVA with Dunn’s multiple comparison post‐test. Correlation analyses were calculated with Spearman’s rho (*r*). *P*‐values were considered significant when < 0.05. Prism v8 (GraphPad Software Inc., San Diego, CA, USA) was used for all statistical analyses. Error bars represent median ± interquartile range; *P ≤ 0.05, **P ≤ 0.01, ***P ≤ 0.001, ***P ≤ 0.0001 and ns = not significant.

## CONFLICT OF INTEREST

MKL received research funding from Bristol Myers Squibb, Takeda, CRISPR therapeutics and Sangamo Inc. for work not related to this study.

## AUTHOR CONTRIBUTIONS

LC designed experiments, acquired, analyzed and interpreted data and wrote the manuscript; KR, EH, BdB, MQW and QH designed experiments, acquired and analyzed data; ST performed CHIPSeq analyses; JKG performed bisulfite sequencing; DJS and MPJW produced TGM and contributed to experiment design; SZ obtained funding and contributed to study concept; RMM and MKL obtained funding and contributed to study concept, design, supervision and critical revision of manuscript.
