# Supplementary material for: Induction of stable human FOXP3+ Tregs by a parasite‐derived TGF‐β mimic
Source: Immunol Cell Biol. 2021 Jun 3;99(8):833–47. doi: 10.1111/imcb.12475 (PMC8453874; doi:10.1111/imcb.12475)
Supplement: Supplementary file 1 [file IMCB-99-833-s001.pdf]

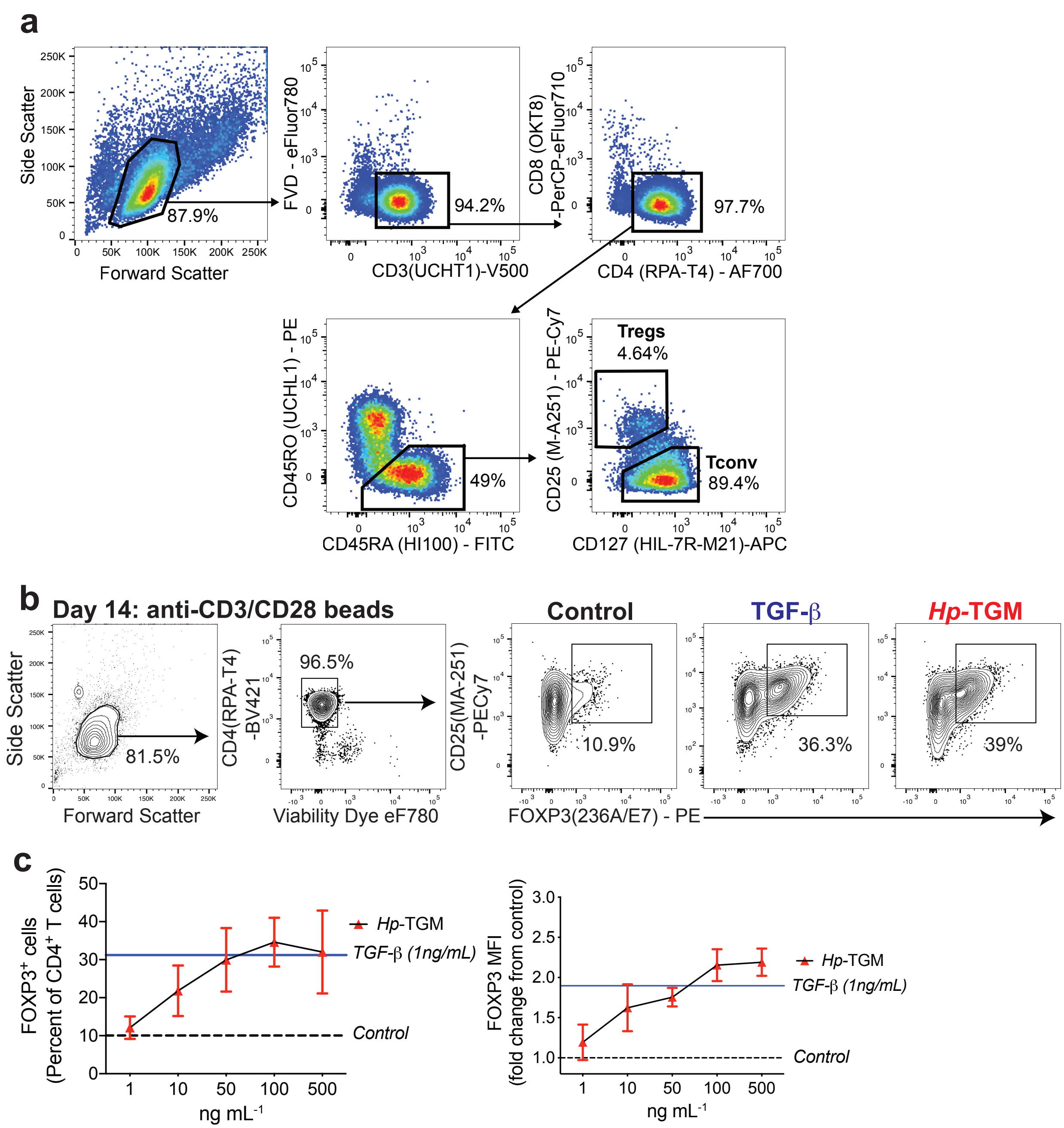

Supplementary figure 1. **Naïve CD4$^+$ T cell isolation and *Hp*-TGM FOXP3 induction with anti-CD3/CD28 bead stimulation**

(a) Gating strategy for isolation of live naïve CD4$^+$ T cells (Tconv) and Tregs. **(b)** Representative data and **(c)** collated data for n = 3 showing induced FOXP3 expression (by percent positive cells and MFI) in control, TGF-β, *Hp*-TGM cultures after 14 days expansion with anti-CD3/CD28 beads at a 1 bead to 4 cells ratio. *Hp*-TGM was used at indicated dose from 1 to 500 ng mL^{-1} (median and interquartile range shown), solid blue line represents median from wells with 1ng mL^{-1} TGF-β added, dotted black represents median from control wells without TGF-β or *Hp*-TGM added.

ChIP-Seq of H3K27Ac (GO analysis)

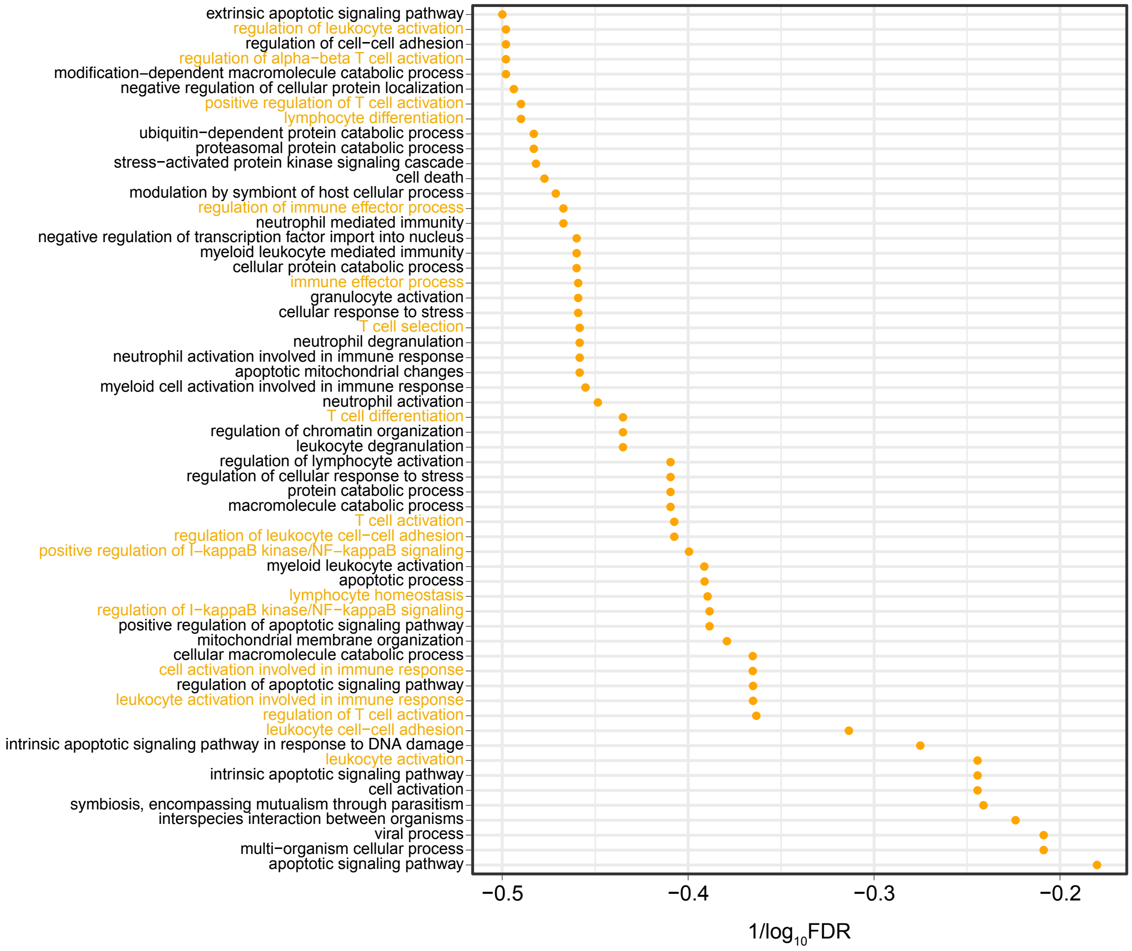

Supplementary figure 2. GO term analysis of ChIP-Seq data

From ChIP-Seq data (n = 2), genes with enriched H3K27Ac marks in *Hp*-TGM-treated samples were analysed for GO terms. Lymphocyte/immune-related GO terms are highlighted in orange.

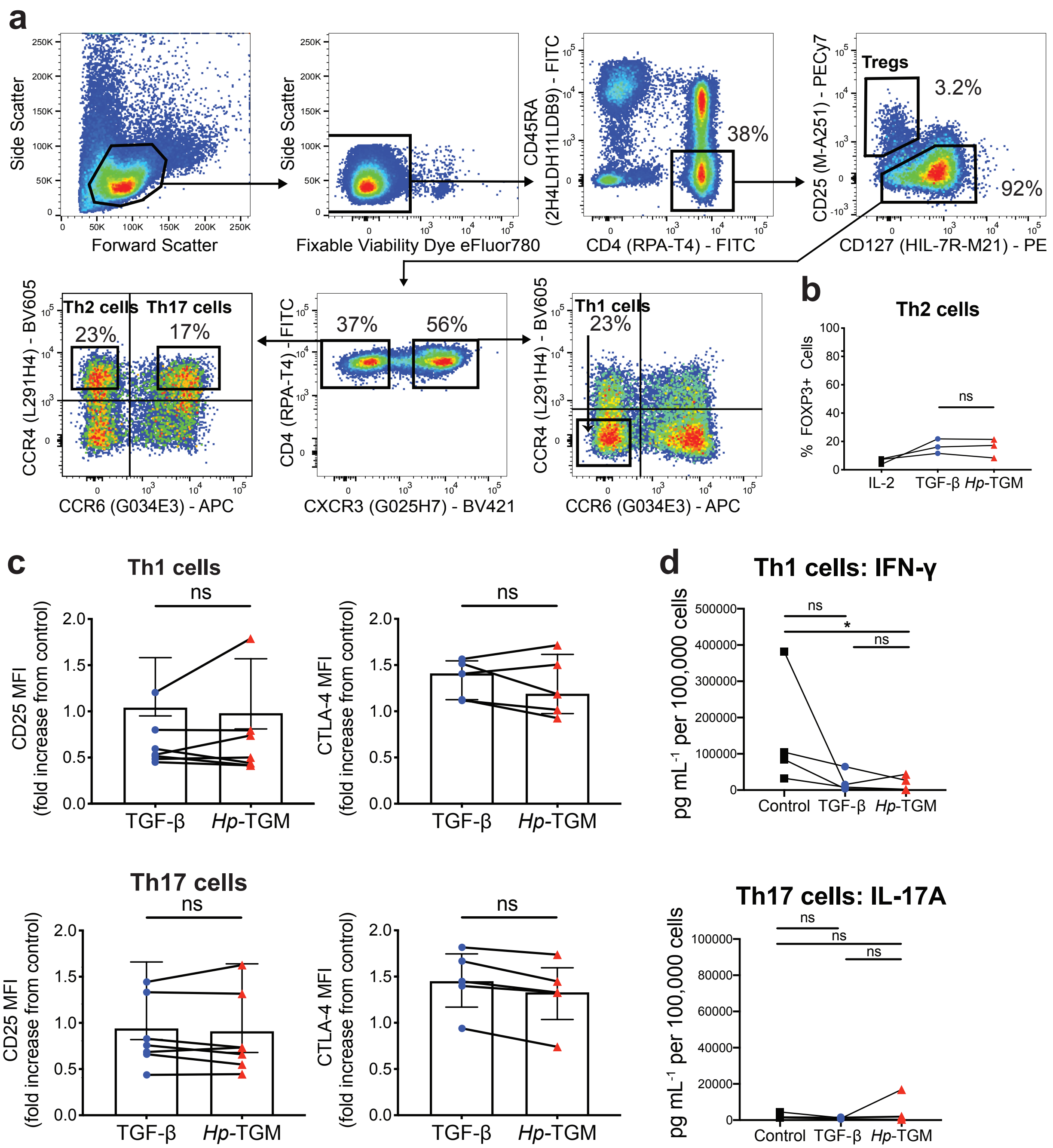

Supplementary figure 3. Isolation of memory CD4^{+} Th cell subsets and *Hp*-TGM induced changes to phenotype and function.

(a) Gating strategy for isolation of live memory CD4^{+} Th2, Th17 and Th1 cells. **(b)** Percent FOXP3^{+} cells in Th2 cell control, TGF-β and *Hp*-TGM cultures after 7 days. After 7 days culture of Th1 cells and Th17 cells we assessed **(c)** CD25 (n = 7) and CTLA4 (n=5) MFI and **(d)** IFN-γ and IL-17A secretion (n = 4). **(e)** The average percent methylation is shown for 8 CpG sites in the TSDR of *FOXP3* for cells from control, TGF-β and *Hp*-TGM cultures of Th1 and Th17 cells (n = 3, all males).
